# The prognostic significance of combined geriatric nutritional risk index and psoas muscle volume in older patients with pancreatic cancer

**DOI:** 10.1186/s12885-021-08094-y

**Published:** 2021-03-31

**Authors:** Teruhisa Sakamoto, Takuki Yagyu, Ei Uchinaka, Kozo Miyatani, Takehiko Hanaki, Kyoichi Kihara, Tomoyuki Matsunaga, Manabu Yamamoto, Naruo Tokuyasu, Soichiro Honjo, Yoshiyuki Fujiwara

**Affiliations:** Department of Surgery, Division of Gastrointestinal and Pediatric Surgery, School of Medicine, Tottori University Faculty of Medicine, 36-1 Nishi-cho, Yonago, 683-8504 Japan

**Keywords:** GNRI, Psoas muscle volume, Pancreatic cancer, Older

## Abstract

**Background:**

The geriatric nutritional risk index (GNRI), originally developed as a nutritional assessment tool to evaluate mortality and morbidity in older hospitalized patients (i.e., those aged ≥65 years), is regarded as a prognostic factor in several cancers. Body composition is also an important consideration when predicting the prognosis of patients with cancer. This study aimed to investigate the relationship between the GNRI and psoas muscle volume (PMV) for survival outcomes in patients with pancreatic cancer.

**Methods:**

This retrospective study evaluated the prognostic significance of the GNRI and PMV in 105 consecutive patients aged ≥65 years who underwent pancreatectomy for histologically confirmed pancreatic cancer. The patients were divided into high (GNRI > 98) and low GNRI groups (GNRI ≤98), and into high (PMV > 61.5 mm^3^/m^3^ for men and 44.1 mm^3^/m^3^ for women) and low PMV (PMV ≤ 61.5 mm^3^/m^3^ for men and 44.1 mm^3^/m^3^ for women) groups.

**Results:**

Both the 5-year overall survival (OS) and recurrence-free survival (RFS) rates were significantly greater among patients in the high GNRI group than among patients in the low GNRI group. Similarly, both the 5-year OS and RFS rates were significantly greater among patients in the high PMV group than among patients in the low PMV group. Patients were stratified into three groups: those with both high GNRI and high PMV; those with either high GNRI or high PMV (but not both); and those with both low GNRI and low PMV. Patients with both low GNRI and low PMV had a worse 5-year OS rate, compared with patients in other groups (*P* <  0.001). The C-index of the combination of the GNRI and PMV for predicting 5-year OS was greater than the C-indices of either the GNRI or PMV alone. Multivariate analysis revealed that the combination of the GNRI and PMV was an independent prognostic factor in patients aged ≥65 years with pancreatic cancer (*P* = 0.003).

**Conclusions:**

The combination of the GNRI and PMV might be useful to predict prognosis in patients aged ≥65 years with pancreatic cancer.

## Background

Various tumor-specific factors and individual factors are reportedly associated with the prognosis of patients with cancer. In recent years, the evaluation of nutritional status and body composition in patients with cancer have received attention in the context of survival outcomes; their potential prognostic values have been reported in various malignant diseases.

The geriatric nutritional risk index (GNRI), first reported by Bouillanne et al., is a novel and well-established objective nutritional assessment tool for the prediction of malnutrition-related risks of mortality and morbidity, including infection and bedsores, in hospitalized older patients (i.e., those aged ≥65 years) [1]. The GNRI consists of two parameters: serum albumin level and body weight; this index has been reported as a prognostic factor in patients with various cancers, such as gastric cancer, hepatocellular carcinoma, and head and neck cancer [2–4].

Body composition is also considered important for predicting survival outcomes. Skeletal muscle wasting (i.e., sarcopenia) contributes to poor prognosis in patients with cancer. This is at least partly because of hypercatabolism related to the exhaustion of skeletal muscle mass in patients with advanced stages of cancer [5–7]. Malnutrition causes secondary sarcopenia, which suggests that the close relationship between nutrition status and body composition may affect survival outcomes in patients with cancer.

Pancreatic cancer is considered one of the most lethal cancers with a 5-year overall survival (OS) rate of < 6% [8]. The prognosis of patients with pancreatic cancer has not been satisfactorily improved despite the development of advanced surgical techniques, perioperative treatment, and progress in terms of systemic therapies (e.g., chemotherapy). Many prognostic factors have been identified in patients with pancreatic cancer. To the best of our knowledge, few studies have investigated the prognostic significance of the GNRI in patients with pancreatic cancer. Furthermore, the impact of the combination of the GNRI and skeletal muscle volume on the prognosis of patients with pancreatic cancer remains unclear. Although skeletal muscle mass and levels of nutritional markers (e.g., serum albumin) generally decrease with age, greater lifestyle-related variations in nutritional status and body composition may be present in older patients, compared with younger patients.

Therefore, this study was performed to evaluate the prognostic significance of the GNRI and to examine the relationship between the GNRI and skeletal muscle volume (specifically, psoas muscle volume [PMV]) in terms of prognosis prediction in patients aged ≥65 years with pancreatic cancer.

## Methods

### Patients

In this study, in accordance with the World Health Organization classification, older individuals were defined as those aged ≥65 years. We retrospectively analyzed the medical records of 105 consecutive older patients with histologically confirmed pancreatic ductal adenocarcinoma who had undergone pancreatectomy with regional lymphadenectomy at our institution between January 2006 and December 2019. The patients enrolled in this study constituted 103 patients with resectable pancreatic cancer and 2 patients with borderline resectable pancreatic cancer. Neoadjuvant chemotherapy was administered to 12 patients; 10 with resectable pancreatic cancer and 2 with borderline resectable pancreatic cancer. We collected all clinicopathological data from their records. All patients in this study were of Japanese ethnicity. The patients’ histopathological findings, such as tumor size, lymph node metastasis, distant metastasis, and histological differentiation, were classified in accordance with the 8th edition of the International Union Against Cancer TNM classification system [9]. No patients had distant metastasis in this study.

### Calculation of geriatric nutritional risk index

The GNRI was calculated using the following formula: GNRI = [14.89 × serum albumin level (g/dL)] + [41.7 × actual body weight/ideal body weight] [1]. The values of serum albumin level and actual body weight in all patients were collected from data that had been obtained on admission (within 1 week prior to surgery). The value of a patient’s actual body weight divided by the ideal body weight was set to 1 when the patient’s weight exceeded ideal body weight [1].

### Measurement and assessment of psoas muscle volume

Total PMV (mm^3^) for each patient was measured by the analysis of preoperative computed tomography images using SYNAPSE VINCENT (Fujifilm, Tokyo, Japan). It was then divided by the cube of height (m^3^) to produce normalized PMV values (mm^3^/m^3^).

### Statistical analysis

Differences between two groups were analyzed using the chi-squared test or Fisher’s exact test for categorical variables; they were analyzed using the Mann–Whitney U test for continuous variables. The 5-year OS and 5-year recurrence-free survival (RFS) rates were estimated by the Kaplan–Meier method; prognostic differences were compared among groups by using the log-rank test.

Receiver operating characteristic analysis was used to calculate the cutoff value of PMV. The concordance index (C-index) was used to evaluate the combination of the GNRI and PMV, and GNRI and PMV, separately, to predict 5-year OS. Univariate and multivariate analyses were performed using Cox proportional hazards models to identify factors with prognostic significance for OS. Variables with *P* <  0.1 in univariate analysis were entered into multivariate analysis. *P* values < 0.05 were considered statistically significant.

All statistical analyses were performed using IBM SPSS Statistics for Windows (version 24; IBM Corp., Armonk, NY, USA).

## Results

The median follow-up interval in this study was 26.6 months (range, 3.3–168.0 months). The mean GNRI of all patients was 97.3 ± 10.2. The mean PMVs were 64.2 ± 13.8 for male patients and 48.4 ± 8.5 for female patients. In accordance with a previous report [1], the cutoff value for the GNRI in this study was set at 98. In contrast, the optimal cutoff values for PMV were evaluated using receiver operating characteristic curve analysis of 5-year OS; these cutoff values were 61.5 mm^3^/m^3^ for men and 44.1 mm^3^/m^3^ for women. Based on the GNRI and PMV cutoff values, the patients were grouped as high GNRI (GNRI > 98, *n* = 55) or low GNRI (GNRI ≤98, *n* = 50), and as high PMV (PMV > 61.5 mm^3^/m^3^ for men and 44.1 mm^3^/m^3^ for women; *n* = 60) or low PMV (PMV ≤ 61.5 mm^3^/m^3^ for men and 44.1 mm^3^/m^3^ for women; *n* = 45).

The relationships between clinicopathological characteristics and the GNRI or PMV are summarized in Table 1. A significant correlation was observed between GNRI and body mass index (BMI), as well as between GNRI and preoperative serum albumin level. Both BMI and preoperative albumin levels were significantly greater in the high GNRI group than in the low GNRI group. There were significant associations of PMV with age, BMI, American Society of Anesthesiologists physical status, preoperative serum albumin level, and adjuvant chemotherapy usage. BMI, preoperative serum albumin level, and adjuvant chemotherapy usage were significantly greater in the high PMV group than in the low PMV group. Moreover, American Society of Anesthesiologists physical status was better in the high PMV group than in the low PMV group, whereas age was significantly younger in the high PMV group than in the low PMV group.

**Table 1:** Clinicopathological characteristics of patients with pancreatic cancer, stratified according to GNRI and PMV

Characteristics	GNRI		*P*	PMV		*P*
	> 98 (*n* = 55)	≤ 98 (*n* = 50)		High group (*n* = 60)	Low group (*n* = 45)	
Age, years, median (range)	73.4 (65–84)	75.4 (66–85)	0.057	73.3 (65–85)	76.2 (67–85)	0.013
Sex (male, %)	26 (56.2%)	33 (66.0%)	0.053	29 (48.3%)	30 (66.7%)	0.061
Body mass index, kg/m^2^, median (range)	22.8 (17.4–30.0)	20.5 (14.0–29.4)	< 0.001	22.8 (17.7–30.0)	20.4 (14.0–28.5)	< 0.001
Tumor size, mm, median (range)	25.6 (10.0–60.0)	27.5 (4.0–60.0)	0.338	25.7 (13.0–60)	27.6 (4.0–60.0)	0.766
Tumor location (head, %)	25 (45.5%)	32 (64.0)	0.057	33 (55.0%)	24 (53.3%)	0.865
Histological grading (G1, %)	30 (54.5%)	27 (54.0%)	0.955	35 (58.3%)	22 (48.9%)	0.336
Resectability status (BR-PC, %)	2 (3.6%)	0 (0.0%)	0.496	1 (1.7%)	1 (2.2%)	1.000
Lymph node metastasis (present, %)	26 (47.3%)	32 (64.0%)	0.085	34 (56.7%)	24 (53.3%)	0.734
ASA-PS (1 or 2, %)	47 (85.5%)	35 (70%)	0.056	51 (85.0%)	31 (68.9%)	0.048
Preoperative albumin, g/dL, median (range)	4.2 (3.8–4.9)	3.7 (2.1–4.3)	< 0.001	4.0 (2.8–4.9)	3.9 (2.1–4.7)	0.033
Preoperative lymphocyte count, median (range)	1495 (140–3800)	1560 (400–6660)	0.947	1512 (140–6660)	1536 (400–3016)	0.763
Preoperative CA19–9, U/mL, median (range)	59.0 (0.7–3221.0)	63.7 (0.7–3270.7)	0.599	59.1 (0.7–3221)	60.8 (0.7–3270.7)	0.591
Neoadjuvant chemotherapy (present, %)	8 (14.5%)	4 (8.0%)	0.366	6 (10.0%)	6 (13.3%)	0.595
Adjuvant chemotherapy (present, %)	32 (60.4%)	27 (55.1%)	0.590	39 (67.2%)	20 (45.5%)	0.027

Abbreviations: *G1* Well-differentiated; *GNRI* Geriatric nutritional risk index; *PMV* Psoas muscle volume; *ASA-PS* American Society of Anesthesiologists physical status; *CA19–9* Carbohydrate antigen 19–9; *BR-PC* Borderline resectable pancreatic cancer

**Fig. 1:**
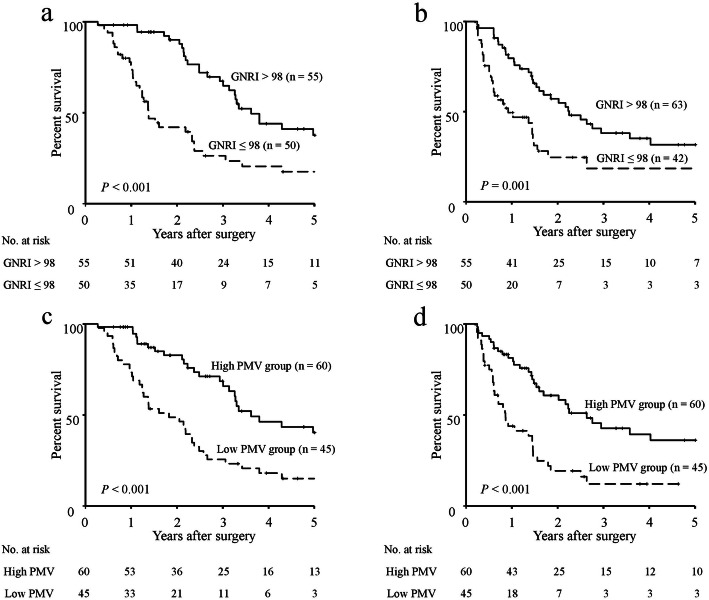
Survival rates in older patients with pancreatic cancer. Five-year overall survival rates (**a**) and recurrence-free survival rates (**b**), compared on the basis of GNRI. Five-year overall survival rates (**c**) and recurrence-free survival rates (**d**), compared on the basis of PMV. Abbreviations: GNRI, geriatric nutritional risk index; PMV, psoas muscle volume

**Fig. 2:**
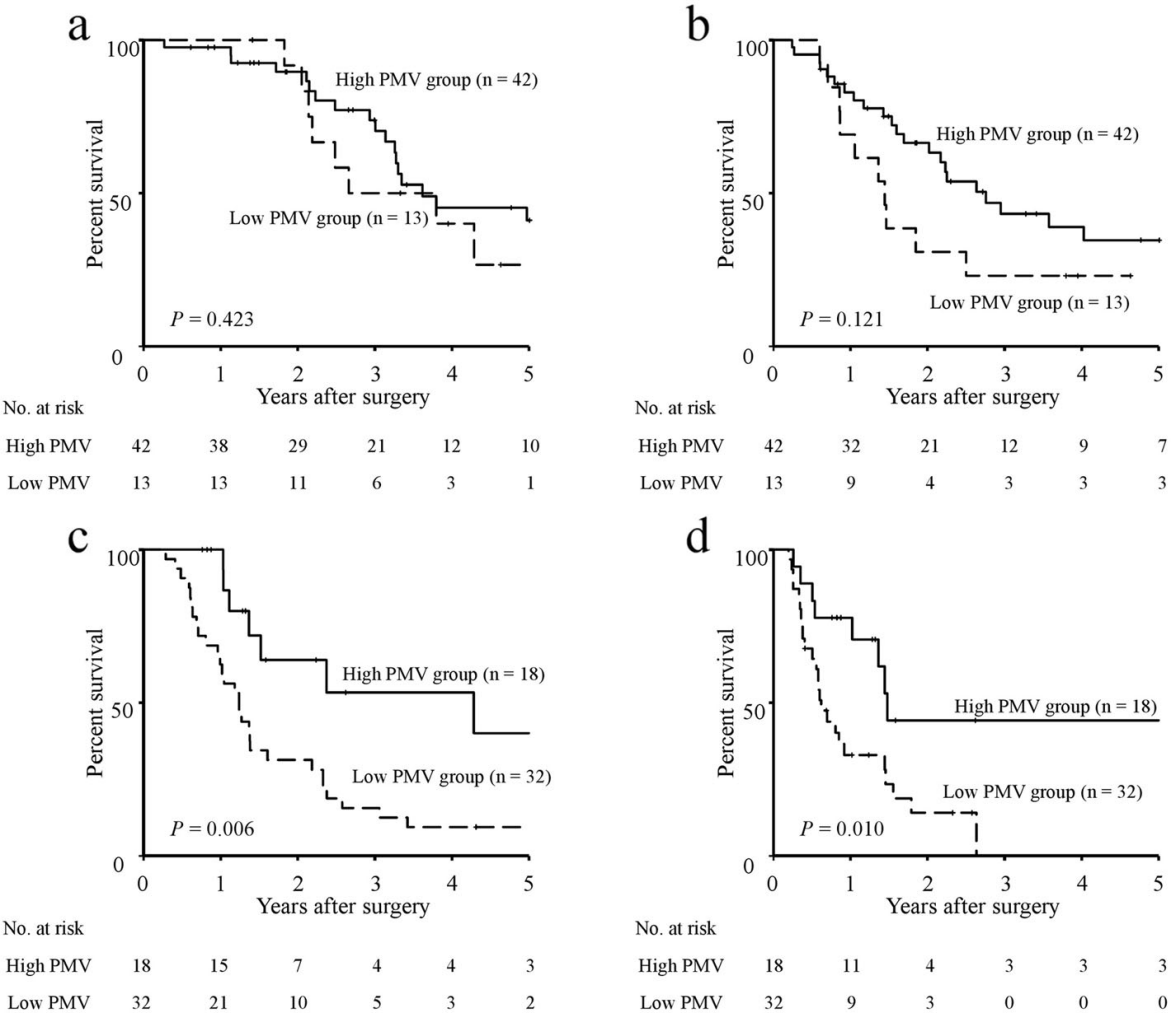
Survival rates in older patients with pancreatic cancer, stratified according to PMV and GNRI. Five-year overall survival rates (**a**) and recurrence-free survival rates (**b**) in patients with high GNRI, compared between low and high PMV. Five-year overall survival rates (**c**) and recurrence-free survival rates (**d**) in patients with low GNRI, compared between low and high PMV. Abbreviations: GNRI, geriatric nutritional risk index; PMV, psoas muscle volume

**Fig. 3:**
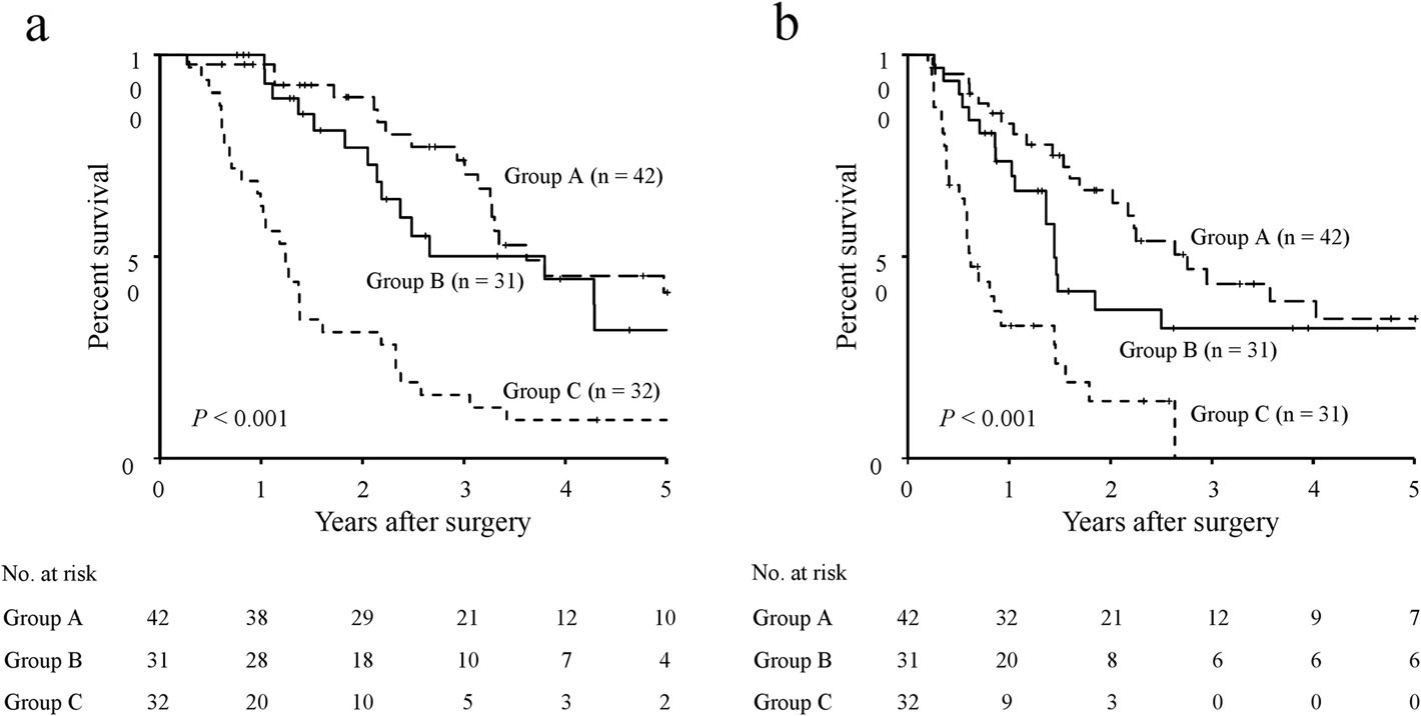
Survival rates in older patients with pancreatic cancer, compared among combinations of PMV and GNRI. Five-year overall survival rates (**a**) and recurrence-free survival rates (**b**) in older patients with pancreatic cancer, compared among patients with distinct combinations of GNRI and PMV. Group A, patients with both high GNRI and high PMV; group B, patients with high GNRI or high PMV (but not both); and group C, patients with both low GNRI and low PMV. Abbreviations: GNRI, geriatric nutritional risk index; PMV, psoas muscle volume

The 5-year OS and RFS rates, stratified according to both the GNRI and PMV, are shown in Fig. 1. The respective 5-year OS and RFS rates were 37.6 and 31.7% in the high GNRI group, whereas they were 17.6 and 18.4% in the low GNRI group. Both 5-year OS and RFS rates were significantly greater in the high GNRI group than in the low GNRI group (OS: *P* <  0.001; RFS, *P* = 0.001; Fig. 1a, b). With respect to PMV, the respective 5-year OS and RFS rates were 40.3 and 36.1% in the high PMV group, whereas they were 14.0 and 12.0% in the low PMV group. The prognoses of patients in the high PMV group were significantly better than those of patients in the low PMV group (OS: *P* <  0.001; RFS: *P* <  0.001; Fig. 1c, d). Analysis stratified according to the GNRI showed no significant differences in 5-year OS and RFS rates between the high PMV and low PMV groups among patients with high GNRI (Fig. 2a, b), whereas the 5-year OS and RFS rates of the low PMV group were significantly lower than those of the high PMV group among patients with low GNRI (OS: *P* = 0.006; RFS: *P* = 0.010; Fig. 2c, d). Accordingly, we stratified patients into three groups: A, patients with both high GNRI and high PMV (*n* = 42); B, patients with high GNRI or high PMV (but not both) (*n* = 31); and C, patients with both low GNRI and low PMV (*n* = 32). Figure 3 shows the 5-year OS and RFS rates for the combination of the GNRI and PMV. The 5-year OS rates were 41.1% in group A, 31.8% in group B, and 9.4% in group C (*P* <  0.001, Fig. 3a). The 5-year RFS rates were 34.6% in group A, 32.2% in group B, and 14.1% in group C (*P* <  0.001, Fig. 3b). The C-index of the combination of the GNRI and PMV for predicting 5-year OS was 0.737, which was greater than the C-index of GNRI (0.686) and the C-index of PMV (0.637).

Multivariate analysis revealed that the combination of the GNRI and PMV was an independent prognostic factor (hazard ratio [HR]: 2.866; 95% confidence interval [CI]: 1.490–5.513; *P* = 0.002), along with BMI (HR: 2.048; CI: 1.105–3.796; *P* = 0.023), tumor size (HR: 2.829; CI: 1.297–6.174; *P* = 0.009), and lymph node metastasis (HR: 2.552; CI: 1.466–4.443; *P* = 0.001) in patients aged ≥65 years who underwent resection of pancreatic cancer (Table 2).

**Table 2:** Potential prognostic factors for overall survival in patients aged ≥65 years with pancreatic cancer

	Univariate analysis			Multivariate analysis		
Variables	HR	95% CI	*P*	HR	95% CI	*P*
Sex (male vs. female)	1.771	1.043–3.006	0.034	1.463	0.814–2.629	0.204
Age (≥ 75 years vs. < 75 years)	1.111	0.675–1.830	0.678			
Body mass index (< 20.5 mm vs. ≥ 20.5 mm)	2.946	1.781–4.873	< 0.001	2.048	1.105–3.796	0.023
Tumor size (≥ 20.0 mm vs. < 20.0 mm)	2.383	1.170–4.855	0.017	2.829	1.297–6.174	0.009
Tumor location (head vs. body and tail)	1.135	0.688–1.874	0.620			
Histological grading for primary tumor (G1 vs. other)	0.667	0.372–1.194	0.173			
Lymph node metastasis (present vs. absent)	2.736	1.604–4.666	< 0.001	2.552	1.466–4.443	0.001
ASA-PS (3 vs. 1 or 2)	1.500	0.837–2.687	0.173			
Preoperative CA19–9 (≥ 152.8 U/mL vs. < 152.8 U/mL)	1.987	1.185–3.330	0.009	1.272	0.749–2.161	0.373
Combination of the GNRI and PMV (Group C vs. Group A and Group B	3.852	2.321–6.393	< 0.001	2.866	1.490–5.513	0.002

Abbreviations: *HR* Hazard ratio; *CI* Confidence interval; *G1* Well-differentiated; *ASA-PS* American Society of Anesthesiologists physical status; *CA19–9* Carbohydrate antigen 19–9; *GNRI* Geriatric nutritional risk index; *PMV* Total psoas muscle volume; Group A, both high GNRI and high PMV; Group B, high GNRI or high PMV (but not both); Group C, both low GNRI and low PMV

## Discussion

This study demonstrated that both GNRI and PMV were associated with the prognosis of older patients (aged ≥65 years) with pancreatic cancer. This study also indicated that the combination of the GNRI and PMV was an independent prognostic factor in older patients with pancreatic cancer; notably, patients with both low GNRI and low PMV had significantly worse prognosis than patients with other combinations of GNRI and PMV.

Malnutrition is closely associated with poor survival in patients with cancer of the digestive system [10–12]. Cancer-related malnutrition is indicative of hypercatabolism promoted by aggressive biological behaviors from cancer [5]. Furthermore, nutritional supplements have been shown to enhance the anti-tumor immune response; early nutritional support can improve patient survival [13, 14]. Therefore, correct evaluation of the nutritional status in patients with cancer is crucial for prognosis prediction because nutritional status may affect cancer progression. Our results indicated that the prognosis of older patients was significantly better in the high GNRI group than in the low GNRI group. This was consistent with the results of previous studies concerning survival outcomes in older patients aged ≥65 with digestive cancers (e.g., esophageal, gastric, and colorectal cancers) [2, 15, 16]. Among several available nutritional assessment tools, the GNRI is regarded as a simple and accurate tool to investigate nutrition-related risks for older patients, with respect to serum albumin level and body weight. Serum albumin synthesized by the liver is considered a marker of both nutrition and inflammation. Albumin levels decrease in the presence of inflammation because of escape to extravascular tissues via inflammation-related enhanced capillary permeability; the production of albumin may also be inhibited by pro-inflammatory mediators such as interleukin-6, interleukin-1, and tumor necrosis factor [17–19]. Additionally, inflammatory cytokines (e.g., transforming growth factor-β and interleukin-6) promote tumorigenesis by enhancing the proliferation, metastasis, and immune escape of tumor cells during chronic inflammation [20, 21]. Hence, the serum albumin level is indicative of tumor progression in the context of cancer-related inflammation. Notably, hypoalbuminemia is a finding consistently associated with adverse outcomes in patients with gastrointestinal cancers, as well as patients with head and neck cancer [22–25]. Several studies have shown that weight loss and malnutrition comprise adverse survival outcomes in patients with cancer [26–28]. These findings could explain the relationship between the GNRI and the prognosis of patients with pancreatic cancer observed in our study.

The GNRI is clearly an easily measurable and readily available nutritional marker in older patients. However, body composition should also be considered to accurately evaluate nutritional status in older patients because there are considerable differences in skeletal muscle volume among these patients. Although substantial loss of muscle mass is generally observed in older patients, skeletal muscle volume in these patients is particularly influenced by their lifestyles and cultural backgrounds. The exhaustion of skeletal muscle is caused by age, as well as malnutrition, inflammatory disease, endocrine changes, and malignancy [29]. Sarcopenia, which comprises the loss of skeletal muscle mass and strength, has been recognized as a prognostic factor in patients aged ≥65 years with cancers of the digestive system [30–32]. There are several methods for measurement of skeletal muscle volumes. Psoas muscle index (PMI) is a comparatively easy method for representing skeletal muscle volume among the measurements used to evaluate sarcopenia; this index is reportedly useful as a prognostic factor in patients with pancreatic cancer [33, 34]. However, PMI is calculated by normalizing the cross-sectional area of the bilateral psoas muscles at the third lumber vertebra to a patient’s height; thus, the PMI may have high measurement error with respect to skeletal muscle volume. Accordingly, we measured the total volume of the psoas muscle in each patient, rather than their PMI. Our findings showed that the PMV was significantly associated with the prognosis of older patients with pancreatic cancer. We thus presumed that the combination of the GNRI and PMV, which are nutritional markers with distinct origins, might better predict prognosis in older patients with pancreatic cancer, compared with prediction using the GNRI or PMV alone. The current study revealed that the C-index of the combination of the GNRI and PMV for predicting 5-year OS was greater than the C-indices of either the GNRI or PMV alone. Finally, low GNRI and low PMV were predictive of significantly worse prognosis, compared with other combinations of the GNRI and PMV, in older patients with pancreatic cancer. These facts suggest that both low GNRI and low PMV accurately represent malnutrition in older patients with pancreatic cancer.

This study had several limitations. First, it was a retrospective cohort study with a small population of only Asian individuals, which might have led to bias and limited the generalizability of the findings. Second, although the cutoff value of the GNRI in this study was set at 98, this value was originally defined as a nutrition-related risk index to predict mortality and morbidity in hospitalized patients aged ≥65 years. Therefore, the optimal cutoff value of the GNRI for OS in older patients with pancreatic cancer remains unclear. Third, the definition of “older” has not been standardized among studies of older patients. Finally, we measured PMV to establish sarcopenia in older patients. However, no consensus has been established concerning methods to measure skeletal muscle volume because body composition—with respect to skeletal muscle mass and strength—in older patients is known to vary according to ethnicity, body size, lifestyle, and cultural background. A large prospective study involving individuals with various ethnicities is necessary to confirm our findings.

## Conclusions

The combination of the GNRI and PMV might be useful to predict prognosis in older patients with pancreatic cancer. Notably, patients with low GNRI and low PMV had the worst pancreatic cancer prognosis. Nutritional management (e.g., nutritional therapy and education) and daily regular exercise might contribute to improved prognosis in older patients with pancreatic cancer.

## Data Availability

The datasets used and analyzed during the current study are available from the corresponding author on reasonable request.

## Abbreviations

GNRI: Geriatric nutritional risk index
PMV: Psoas muscle volume
OS: Overall survival
RFS: Recurrence-free survival
AUC: Area under the curve
BMI: Body mass index
PMI: Psoas muscle index
